# Interatomic Coulombic decay cascades in multiply excited neon clusters

**DOI:** 10.1038/ncomms13477

**Published:** 2016-12-05

**Authors:** K. Nagaya, D. Iablonskyi, N. V. Golubev, K. Matsunami, H. Fukuzawa, K. Motomura, T. Nishiyama, T. Sakai, T. Tachibana, S. Mondal, S. Wada, K. C. Prince, C. Callegari, C. Miron, N. Saito, M. Yabashi, Ph. V. Demekhin, L. S. Cederbaum, A. I. Kuleff, M. Yao, K. Ueda

**Affiliations:** 1 Department of Physics, Graduate School of Science, Kyoto University, 606-8502 Kyoto, Japan; 2 RIKEN SPring-8 Center, 679-5148 Hyogo, Japan; 3 Institute of Multidisciplinary Research for Advanced Materials, Tohoku University, 980-8577 Sendai, Japan; 4 Theoretische Chemie, Physikalisch-Chemisches Institut, Universität Heidelberg, D-69120 Heidelberg, Germany; 5 Department of Physical Science, Hiroshima University, 739-8526 Higashi-Hiroshima, Japan; 6 Elettra-Sincrotrone Trieste, Basovizza, Trieste I-34149, Italy; 7 Synchrotron SOLEIL, L'Orme des Merisiers, Saint-Aubin, BP 48, FR-91192 Gif-sur-Yvette Cedex, France; 8 Extreme Light Infrastructure-Nuclear Physics (ELI-NP), ‘Horia Hulubei' National Institute for Physics and Nuclear Engineering, RO-077125 Măgurele, Jud. Ilfov, Romania; 9 National Metrology Institute of Japan, AIST, 305-8568 Tsukuba, Japan; 10 Theoretische Atom- und Molekülphysik, Institut für Physik und CINSaT, Universität Kassel, D-34132 Kassel, Germany; 11 Deceased

## Abstract

In high-intensity laser light, matter can be ionized by direct multiphoton absorption even at photon energies below the ionization threshold. However on tuning the laser to the lowest resonant transition, the system becomes multiply excited, and more efficient, indirect ionization pathways become operative. These mechanisms are known as interatomic Coulombic decay (ICD), where one of the species de-excites to its ground state, transferring its energy to ionize another excited species. Here we show that on tuning to a higher resonant transition, a previously unknown type of interatomic Coulombic decay, intra-Rydberg ICD occurs. In it, de-excitation of an atom to a close-lying Rydberg state leads to electron emission from another neighbouring Rydberg atom. Moreover, systems multiply excited to higher Rydberg states will decay by a cascade of such processes, producing even more ions. The intra-Rydberg ICD and cascades are expected to be ubiquitous in weakly-bound systems exposed to high-intensity resonant radiation.

## Abstract (web summary)

Interatomic Coulombic decay (ICD) is a relaxation of an atom in a weakly bound environment by the transfer of excess energy to ionize the neighbouring atom. Here the authors observe intra-Rydberg ICD in neon clusters, which is a decay that involves the ionization of Rydberg atoms in the cluster.

## Graphical abstract

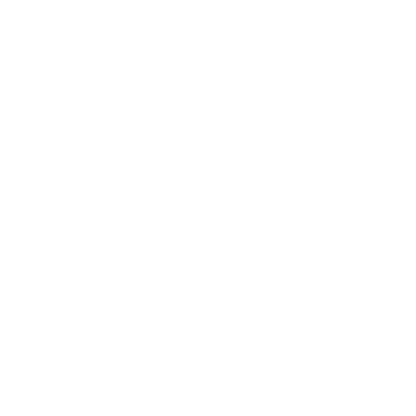

When an inner-valence ionized atom or molecule is surrounded by other atoms or molecules, the energy released via the electronic relaxation of the ionized species can be transferred to a neighbouring atom or molecule, which uses the released energy to emit one of its electrons. This process is called interatomic/intermolecular Coulombic decay (ICD)^1^. Since the first prediction in 1997 (ref. 1), ICD has been extensively studied both theoretically and experimentally in a variety of weakly bound systems (for recent reviews, see refs 2, 3). It is worth noting that ICD has also been observed in molecular water clusters, suggesting that ICD may also occur in water-abundant biological systems^4^,^5^ and be an important source of genotoxic low-energy electrons and radical cations.

Intriguingly it has been shown theoretically and verified experimentally that ICD is not an isolated phenomenon, but might be part of complex cascade relaxation mechanisms, occurring for instance following an Auger decay process^6^,^7^,^8^,^9^,^10^,^11^,^12^,^13^,^14^. To understand relaxation mechanisms, it is necessary to be aware of all possible channels and determine their significance, and in the present work, we investigated this subject.

In the regime of high-intensity radiation, Kuleff *et al*.^15^ proposed an ICD mechanism in which two neighbouring species are resonantly photo-excited to low-lying excited states, and the de-excitation of one of them leads to the ionization of the other via an ultrafast energy transfer. It was argued that this process could be an additional important ion-production mechanism in clusters exposed to intense resonant light, largely dominating the direct multi-photon ionization of clusters' constituents at moderate intensities. Recent experimental studies of large helium droplets excited to low-lying states further supported the high efficiency of this variant of the decay processes^16^,^17^. What happens when the cluster constituents are brought to higher excited states?

In this article, we show that in this case different relaxation dynamics take place. We report the observation of a new type of ICD, as well as of a cascade of ICD processes. For ease of analysis and interpretation, we chose to study neon clusters. Ne clusters containing about 5,000 atoms were irradiated by free electron laser (FEL) pulses of 61 nm wavelength, corresponding to the *2p*^6^→*2p*^5^*3d* atomic-like transition in the cluster^18^. The FEL pulse produces a multiply excited cluster which can relax either via ICD in which one of the excited *3d* electrons recombines with the *2p* hole, as shown in Fig. 1a, or with a much slower rate, by fluorescence. The present experiment, however, clearly demonstrated that this ‘direct' ICD channel is significantly suppressed and instead, ICD transitions that lead to the final state *2p*^5^*3s* state (see Fig. 1b) are dominant. Hereafter, we will refer to these transitions as ‘intra-Rydberg' ICD. We have also observed secondary ICD processes (ICD cascades) resulting from the intermediate excited states such as *2p*^5^*3s* populated by the primary ICD processes (see Fig. 1c). We note that similar processes have been studied in a completely different regime and form of matter, namely Rydberg gases, and shown to be responsible for the so-called avalanche ionization phenomenon and the creation of ultracold plasma^19^,^20^,^21^,^22^. In Rydberg gases, however, collisions are recognized as a prerequisite for the ionizing mechanism, while in the van der Waals clusters studied by us the energy transfer between excited atoms is so efficient, due to the much smaller interatomic distances, that collisions (although not excluded) are not necessary for producing electrons and ions. Our study on clusters complements that on Rydberg gases and makes clear the importance of cascades in condensed matter.

## Results

### Experimental approach

The experiments were performed at the SPring-8 Compact SASE Source test accelerator in Japan^23^. Intense EUV-FEL pulses at 20.3 eV photon energy (bandwidth of 0.3 eV full-width at half-maximum (FWHM)) and of 30 fs duration were focused on a Ne cluster beam. The kinetic energy distribution of the emitted electrons was measured with a velocity map imaging (VMI) spectrometer (see Fig. 2a, Methods and ref. 24). Electron velocity map images for Ne clusters and atoms are shown in Fig. 2. The photon energy of 20.3 eV corresponds to the resonant excitation energy in a cluster of ground-state Ne atoms to the *2p*^5^ *3d* states. Despite the high FEL intensity, it is evident from Fig. 2 that the FEL pulse does not cause any significant electron emission from isolated atoms despite its high intensity (∼5.3 × 10^12^ W cm^−2^). The situation changes dramatically for clusters, where a strong electron signal is produced already at moderate FEL intensities (∼4.5 × 10^11^ W cm^−2^) due to the ICD processes.

### Electron emission spectra of neon clusters and assignment

Electron spectra from Ne clusters are shown in Fig. 3. On top of the well-known exponential thermal distribution of the electrons^25^, stemming from the increase of the cluster potential by charge accumulation and nanoplasma formation, we also observe distinct structures at about 1.8 and 11 eV that become more pronounced with increasing FEL intensity.

**Figure 1:**
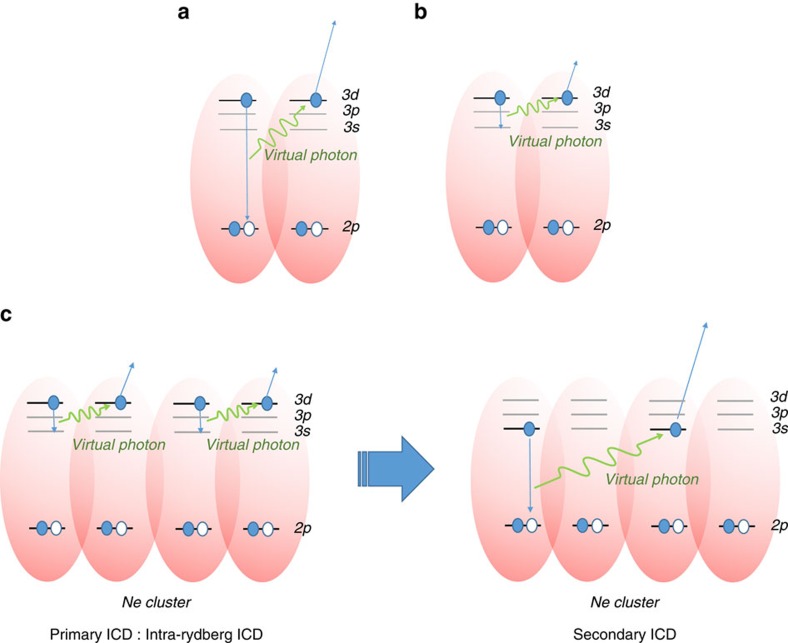
Schematic diagram of Interatomic Coulombic Decay transitions from resonant excited states. (**a**) Schematic diagram of the direct Interatomic Coulombic Decay (ICD) process: Ne*(*2p*^5^*3d*)+Ne*(*2p*^5^*3d*)→Ne^+^(*2p*^5^)+Ne(*2p*^6^)+e^ICD^, which can be abbreviated as *3d*+*3d*→*2p*+e^ICD^. Here one excited neon atom, Ne*(*2p*^5^*3d*), returns to its ground state (Ne(*2p*^6^)) and the other neon atom is ionized by using the energy transferred by a virtual photon exchange. (**b**) Intra-Rydberg ICD: *3d*+*3d*→*3s*+e^ICD^. In this process, one Ne*(*2p*^5^*3d*) undergoes a transition to a lower excited state, Ne*(*2p*^5^*3s*), and the other one is ionized. Note that excited neon atoms remain in the neon cluster after intra-Rydberg ICD. (**c**) ICD cascade: after an intra-Rydberg ICD, the excited species thus produced continue to decay by secondary ICD processes either among themselves, or with other neighbouring excited species. Note that such an ICD cascade produces more ions compared with the case when the multiply-excited cluster would have decayed only by direct ICD processes.

**Figure 2:**
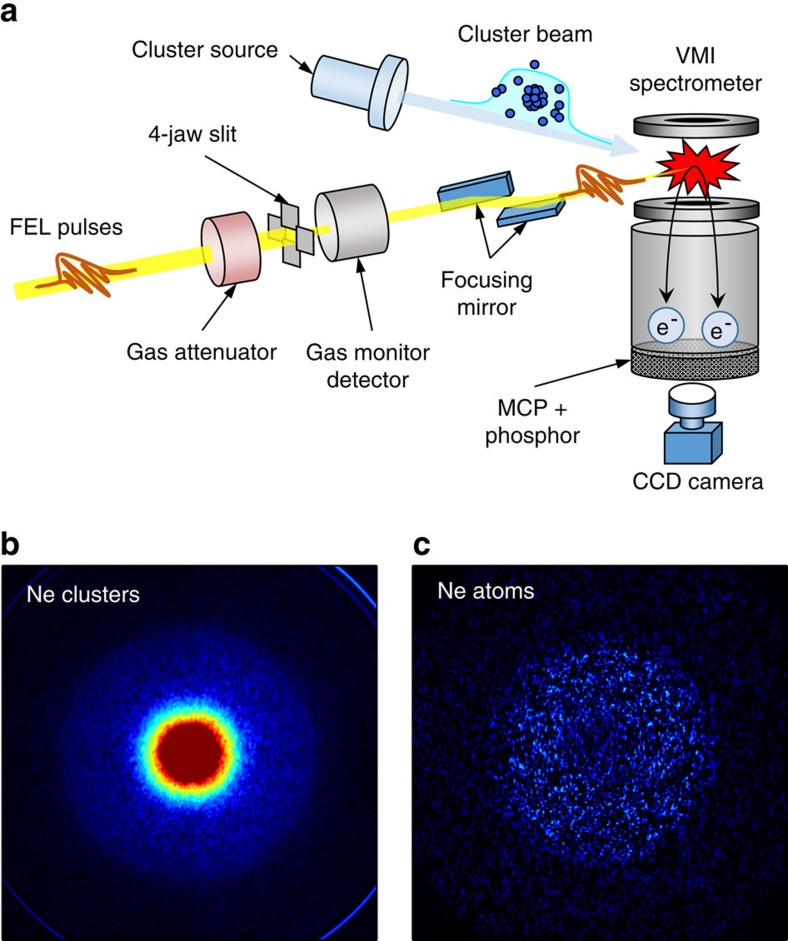
Experimental set-up and raw experimental data. (**a**) Schematic drawing of experimental set-up (see Methods for details). (**b**) Velocity map images of electron distributions of Ne clusters. (**c**) Velocity map images of electron distributions of Ne atoms. The samples were exposed to 20.3 eV Free Electron Laser FEL radiation at ∼4.5 × 10^11^ and ∼5.3 × 10^12^ W cm^−2^ (FEL) intensities, respectively. The VMI spectrometer voltages were set to detect electrons with kinetic energy up to 25 eV. Even at an order of magnitude lower intensity, we observe a much stronger electron signal in the case of clusters, showing the high efficiency of the Interatomic Coulombic Decay (ICD) as an electron-production process.

Before assigning the observed structures, let us first consider the possible ICD transitions in Ne clusters under the present conditions, using the atomic energy level data for Ne (ref. 26). Table 1 summarizes all energetically allowed transitions. Apart from the *3d* to *2p* transition, the *3d* to *3s* transition in one Ne atom is able to eject a *3d* electron from a neighbouring excited atom. This intra-Rydberg ICD process can be viewed as a ‘super Coster-Kronig' transition since all participating electrons occupy *n*=3 shells and, as we will see, dominates the other ICD processes. Moreover, the Ne*(*2p*^5^*3s*) produced by the intra-Rydberg ICD processes can continue to decay by ionizing neighbouring Ne*(*2p*^5^*3d*) or Ne*(*2p*^5^*3s*) (see Fig. 1c).

**Figure 3:**
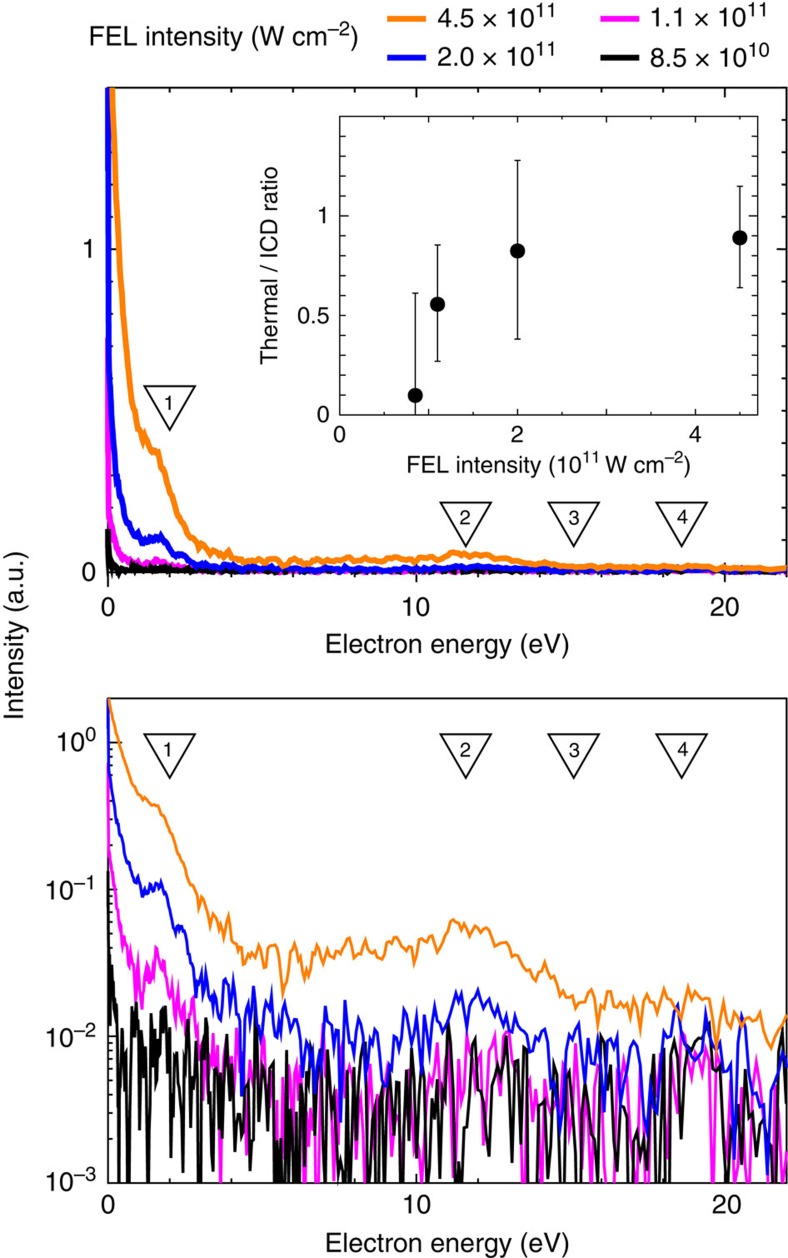
Electron emission spectra of Ne clusters. Electron emission spectra of Ne clusters (N∼5000) irradiated by 20.3 eV photons at different Free Electron Laser (FEL) intensities. The *y*-axis scale of lower panel is logarithmic. Inset: The intensity ratio of thermal electrons to Interatomic Coulombic Decay (ICD) electrons is plotted as filled circles with error bars. Error bars represent the uncertainty in estimated intensity ratio. The triangles indicate the ICD electron energies calculated using the atomic energy levels, labelled as in Table 1.

As we see from Table 1, an interacting pair of Ne*(*2p*^5^*3d*) atoms within a cluster can undergo 4 different ICD transitions (2 primary and 2 secondary) emitting electrons with different energies. These energies, obtained using atomic data, are shown with triangles in Fig. 3. The comparison of these ICD electron energies with the electron spectra suggests that the 1.8 eV peak could be attributed to the *3d*+*3d*→*3s*+e^ICD^ decay route, while the structure around 11 eV could stem from the secondary ICD process: *3s*+*3s*→*2p*+e^ICD^. The transition *3s*+*3d*→*2p*+e^ICD^ emitting electrons with ∼15 eV, and the ‘direct' *3d*+*3d*→*2p*+e^ICD^ transition (∼18.6 eV) are not visible in the experimental spectra, as a consequence of their much lower decay rates (see below).

We note a small discrepancy between the estimated energies of the ICD electrons and the observed peak positions. One reason for the discrepancy could be the interatomic interaction potentials within the cluster that are not included in the estimation using atomic energy levels. Also, the energy of the emitted electrons may be shifted due to the increase of the cluster Coulomb field, as we discuss later.

### Theoretical modelling of the ICD transition rates

To validate our interpretation of the measured electron spectra, we performed numerical simulations based on a system of rate equations. The system of equations describes the time evolution of the population of the excited atoms, which can decay pair-wise via an energetically allowed ICD transition with the corresponding rate. To obtain the rate of each possible ICD transition, we made use of the asymptotic formula for the ICD width Γ (derived in ref. 15) which involves the oscillator strength *f* of the de-excitation transition in one of the atoms in the pair, and the cross-section *σ* to ionize the other excited electron in the other atom with the energy *ω* released by the de-excitation transition:


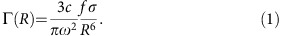


In the above expression, given in atomic units, *c* denotes the speed of light and *R* is the distance between the excited atoms. For all possible ICD transitions, the required atomic quantities (oscillator strengths and ionization cross-sections) were computed at the Hartree-Fock level.

**Figure 4:**
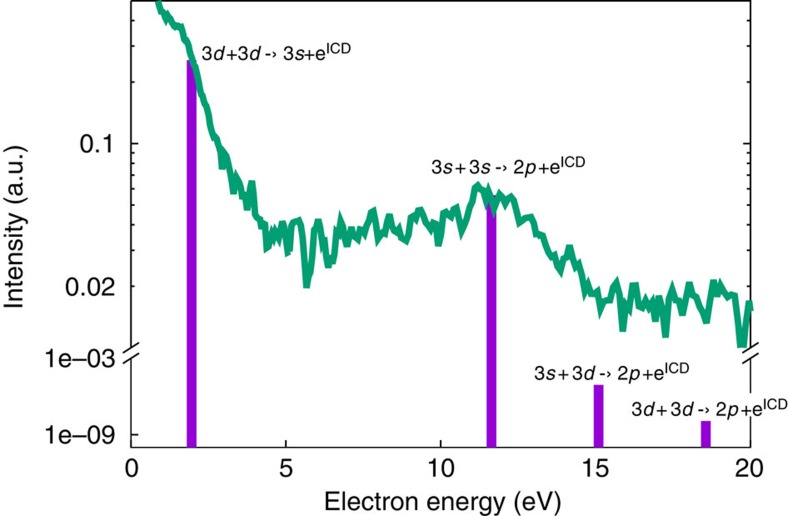
Comparison between experimental data and theoretical simulations. Electron spectra (curves) and computations based on the solution of rate equations (vertical bars) given on a logarithmic scale. The electron spectra shown represent the experimental data recorded at 4.5 × 10^11^ W cm^−2^. Note the break on the *y*-axis and the different scales below and above it. All Interatomic Coulombic Decay (ICD) transitions are also denoted. The computed data are normalized to the measured ones by the intensity of the peak at ∼12 eV. An exponential function accounting for the thermal distribution of the electron emission is extracted from the experimental spectra and added to the theoretical results, but not the experimental background.

The rate equations were solved for the number of electrons emitted in all possible ICD channels along the cascade (see Methods). The results are shown as a histogram in Fig. 4 together with the experimental data. The model cannot predict absolute intensities, but only relative ones, assuming all excited pairs have decayed and all emitted electrons have reached the detector with their initial energies. The model is also not sensitive to the initial concentration of Ne*(*2p*^5^*3d*) and, therefore, to the FEL pulse intensity. That is why, to compare with the experimental spectrum, an exponential function accounting for the thermal distribution of the electron emission has been extracted from the experimental curve and added to the theoretical results, and the resulting data have been normalized to the intensity of the measured peak at ∼11.6 eV. We see that despite the simplicity of the model, it correctly describes the experimental observations: two strong peaks at ∼2 and ∼11.6 eV are predicted with an intensity ratio of approximately 2:1, while the transitions at ∼15.1 and ∼18.6 eV have very low rates and are thus not observed experimentally. The high-intensity of the line at ∼2 eV is due to the high decay rate of the *3d*+*3d*→*3s*+e^ICD^ transition which nearly completely suppresses the ‘direct' *3d*+*3d*→*2p*+e^ICD^ process (see column 6 in Table 1). According to our calculations, the *3s*+*3s*→*2p*+e^ICD^ transition is also much more efficient compared with the other secondary ICD transitions and, therefore, is the dominant decay mode in the second stage of the decay giving rise to the peak at ∼11.6 eV. We note that relaxing via a two-step ICD cascade produces up to 1.5 times more ions compared with the direct ICD transition, the upper limit corresponding to the case when all atoms that have relaxed to their *3s* excited state in the first ICD step decay pair-wise in a second ICD transition.

## Discussion

Let us discuss the intensity and the shape of the peak at around 11.6 eV. We see that the peak becomes more prominent at higher FEL intensities, has an asymmetric shape, and a developing plateau on the low-energy side. Bostedt *et al*.^27^ reported a similar feature in the photoelectron spectra of small argon clusters recorded at 38.7 eV. They suggested a multi-step ionization model, where sequential 1-photon–1-electron photoionization is assumed, and the plateau is attributed to the deceleration of the electrons by the developing attractive Coulomb potential of the cluster ion. In the present experiment, the *3s*+*3s*→*2p*+e^ICD^ process can take place after at least two primary ICD transitions have produced two positive charges in the cluster. Therefore, in the multi-step ICD process one can expect a deceleration of the ICD electrons by the accumulation of positive ions along the *3d*→*3s*→*2p* cascade, shifting the energies of the emitted ICD-electrons and thus forming a plateau in the electron spectrum. In addition, the increase of the cluster potential by charge accumulation would result in trapping the low-energy ICD electrons and thus nanoplasma formation. The intensity ratio of thermal electrons to ICD electrons increases with the increase in the FEL intensity up to 2.0 × 10^11^ W cm^−2^ and becomes approximately constant in the FEL intensity range of 2.0 × 10^11^–4.5 × 10^11^ W cm^−2,^, as depicted in the inset of Fig. 3, suggesting that the source of thermal electrons could be trapping of the low-energy electrons created by the intra-Rydberg ICD by the charged cluster created by the ICD cascade.

Before concluding, a brief account will be given of the comparison between the present work and previously reported ones^16^,^17^,^24^. LaForge *et al*.^16^ and Ovcharenko *et al*.^17^ reported results on helium droplets at the resonance derived from the atomic He *1s2p* state. The energy of de-excitation from *1s2p* to *1s*^*2*^s is, however, not sufficient to ionize another *1s2p* excited atom and thus is transferred only to the nuclear motion. In the present work, a novel phenomenon is observed for multiple excitation to higher Rydberg states, where the de-excitation energy of the intra-Rydberg transition can be transferred to another Rydberg atom and ionize it. Ovcharenko *et al*. clearly identified inelastic scattering of the energetic direct ICD electrons by the excited atoms and called it collective autoionization since more than two excited atoms are involved. In the present experiment, however, we could not identify such processes because of much lower density of excited atoms in the cluster, due to lower FEL intensity by more than one order of magnitude and lower inelastic scattering cross sections. The work of Yase *et al*. on the same excitation of neon clusters^24^ may be more closely related to the present work. However, that work focused on high power (10^13^–10^14^ W cm^−2^) and observed different physics. There, the excited *3d* electrons were instantaneously delocalized and a nanoplasma was formed. As a result ICD was suppressed. In the present work, nanoplasma formation was significantly reduced by reducing FEL intensity and thus ICD could be observed.

In conclusion, in the electron emission spectra of Ne clusters exposed to intense EUV-FEL pulses producing multiple excitations to *3d* levels, we have identified a novel intra-Rydberg ICD mechanism occurring in the same electronic shell, which is much more efficient than the ICD to the ground state. Moreover, multiple population of higher Rydberg states in a cluster also triggers a very efficient cascade of ICDs in a step-wise de-excitation to the ground state, producing in that way more ions. A two-step cascade may produce up to 50% more ions compared with a single step relaxation. We note that, like all previously discovered ICD processes, these intra-Rydberg ICD and ICD cascades are expected to be general phenomena occurring in any weakly bound media (notably: water) exposed to intense light that is resonant with a higher-lying excited state of its constituents.

## Methods

### Experimental methods

The experiments were performed at the SPring-8 Compact SASE Source (SCSS) test accelerator in Japan^23^. Intense EUV-FEL pulses were focused on a Ne cluster beam, and the kinetic energy distribution of emitted electrons was measured with a velocity map imaging (VMI) spectrometer^28^. The Ne clusters were prepared by adiabatic expansion of Ne gas through a 0.25 mm nozzle^29^. The Ne stagnation pressure was 10.8 bar, and the nozzle was cooled to 80 K using liquid nitrogen. The average cluster sizes 〈N〉 were estimated to be about 5,000 atoms according to the well-known scaling law^30^,^31^. The FEL light source provided EUV pulses with a photon energy of 20.3 eV (bandwidth was 0.3 eV FWHM) and a duration of 30 fs at a repetition rate of 30 Hz. The FEL beam was steered by two upstream SiC plane mirrors, passed a gas attenuator, a 4-jaw slit, and a gas monitor detector before entering the pre-focusing system of the beam line. The FEL intensity was reduced by the 4-jaw slit down to 5% of full power and was adjusted by the gas attenuator. The skimmed FEL beam with a mean spot size of 14 μm (FWHM) was focused onto the cluster beam at the center of the VMI spectrometer. The emitted electrons were accelerated toward a position-sensitive detector consisting of a set of micro channel plates (MCPs) followed by a phosphor screen, and recorded using a CCD camera synchronized to the arrival of the FEL pulse in the interaction chamber. The measured two-dimensional (2D) projection allows for the reconstruction of the three-dimensional (3D) momentum distribution of the ejected electrons using the pBASEX algorithm^32^. The energy scale of the electron spectrum was calibrated using the 3p photo-line of atomic argon (*IP*=15.76 eV) with an energy resolution of 5%.

### Theoretical modelling

The present calculations were performed by solving a system of coupled rate equations that describes the population of the excited atoms decaying pair-wise by a cascade of ICD processes. For extracting the rate constant for each type of ICD transition, the Ne cluster was represented by a face-centered cubic system with a lattice constant of 4.4 Å (corresponding to a nearest-neighbor distance of 3.1 Å) filling a sphere with a radius of 29.6 Å, resulting in a cluster of 5,017 neon atoms. Assuming that the FEL pulse can excite about 10% of the atoms in the cluster, the total decay rate (width) of each transition was obtained as the sum of the partial ICD widths, Γ, of all possible pairs of 500 randomly-distributed excited neon atoms estimated via equation (1). The latter was derived by expanding the Coulomb transition matrix element between the ICD initial and final electronic states of the dimer in a multipole series, and keeping only the dipole–dipole term^15^,^33^. In this asymptotic approximation, the ICD width is the product of the transition probabilities for the radiative de-excitation in one of the atoms and for the photoionization of the other one. Those transitions are characterized by the excitation oscillator strength *f* and by the photoionization cross-section *σ* which were computed at the Hartree-Fock level. We note that although within this approximation the radiative transition *3d*→*3s* is dipole-forbidden, the ICD process *3d*+*3d*→*3s*+e^ICD^ is allowed, since the outgoing electron carries the angular momentum required for the conservation of the total momentum in the Coulomb matrix element. Moreover, in a large cluster the orbital momentum is no longer a good quantum number as the atomic orbitals overlap locally to form mixed states. Thus, a strict distinction between *p, d,* and so on. Rydberg state is nearly impossible. The important difference is between intra- and inter-shell transitions. That is why, in order to estimate the ICD width for *3d*+*3d*→*3s*+e^ICD^ transition on the same level of theory, the oscillator strength of the atomic dipole-allowed *3d*→*3p* transition was utilized in equation (1). This is a rough assumption, but the model is not very sensitive to the exact value of the oscillator strength. It can be a few orders of magnitude smaller and will still quench the *3d*+*3d*→*2p*+e^ICD^ process. This is a result of the other quantities entering in equation (1), the ionization cross-section σ and the virtual photon energy ω, which also largely favour the *3d*+*3d*→*3s*+e^ICD^ transition. The ionization cross-section for removing a *3d* electron with a 2 eV photon (corresponding to the *3d*→*3s* transition) is about 3 orders of magnitude larger than the cross-section for removing the same electron with a 19 eV photon (corresponding to the *3d*→*2p* transition). In addition, the decay rate depends as 1/*ω*^2^ on the virtual-photon energy.

We note also that since the model does not take the cluster dynamics into account and assumes that all excited pairs have decayed by ICD to produce electrons with the respective energies, it is not sensitive to the initial population of Ne*(*2p*^5^*3d*) in the cluster and, therefore, to the FEL intensity.

### Data availability

The data that support the findings of this study are available from the corresponding author on reasonable request.

## Additional information

**How to cite this article:** Nagaya, K. *et al*. Interatomic Coulombic decay cascades in multiply excited neon clusters. *Nat. Commun.* **7,** 13477 doi: 10.1038/ncomms13477 (2016).

**Publisher's note:** Springer Nature remains neutral with regard to jurisdictional claims in published maps and institutional affiliations.

## Figures and Tables

**Table 1:** Electron energies of possible transitions.

**Label**	**Primary ICD Process (Intra-Rydberg ICD)**	**Secondary ICD Process (Cascade ICD)**	**Estimated ICD electron energy**	**Observed ICD electron energy**	**Estimated ICD lifetime**
1	*3d*+*3d*→*2p*+e^ICD^		18.6 eV	–	87.8 ns
2	*3d*+*3d*→*3s*+e^ICD^		2 eV	1.8 eV	9.2 fs
3		*3s*+*3s*→*2p*+e^ICD^	11.6 eV	11 eV	18.3 ps
4		*3s*+*3d*→*2p*+e^ICD^	15.1 eV	–	0.5 ns

Estimated and observed ICD electron energies for the possible ICD processes following multiple *2p*→*3d* excitations in Ne clusters. The ICD lifetimes are estimated for a pair of excited neon atoms at 3.15 Å from each other, using equation (1).
